# Impact on decision making framework for medicine purchasing in Chinese public hospital decision-making: determining the value of five dipeptidyl peptidase 4 (DPP-4) inhibitors

**DOI:** 10.1186/s12913-021-06827-0

**Published:** 2021-08-12

**Authors:** Yun Bao, Bei Gao, Min Meng, Bin Ge, Yan Yang, Chunchun Ding, Bingyin Shi, Limin Tian

**Affiliations:** 1 Institute of Clinical Research and Evidence Based Medicine, Gansu Provincial Hospital, Gansu, China; 2 Department of Pharmacy, Gansu Provincial Hospital, Gansu, China; 3 Department of Endocrinology and Metabolism, The First Affiliated Hospital of Xi’an Jiaotong University, No.277 Yanta West Road, Xi’an, Shaanxi China; 4 Department of Endocrinology, Gansu Provincial Hospital, Lanzhou, China; 5 Clinical Research Center for Metabolic Diseases, No. 204, West Road of Donggang Street, Lanzhou, Gansu China

**Keywords:** Evidence and Value: Impact on Decision Making (EVIDEM) framework, Multi-criteria decision analysis (MCDA), Hospital-based health technology assessment (HB-HTA)

## Abstract

**Background:**

Medicine purchasing in Chinese public hospitals is decided by the hospital Pharmacy Management Committee (PMC), that is complex, subjective and requires efficient approaches to ensure transparency and consistency for the factors being considered. This study aimed to use the Evidence and Value: Impact on Decision Making (EVIDEM) framework to assess medicine in these hospitals. In this study anti-diabetic drugs DPP-4 inhibitors, which work by inhibiting the activation of the Dipeptidyl Peptidase 4 (DPP-4) inhibitors, were appraised.

**Methods:**

Following EVIDEM methodology (EVIDEM-10th), we convened an appraisal group and asked each individual to express their perspectives by assigning weights to each criterion. A systematic literature search for information of each criterion of five DPP-4 inhibitors was completed. Then the appraisal group scored for each criterion of the five DPP-4 inhibitors. The estimated value of the five DPP-4 inhibitors was obtained by Multi-Criteria Decision Analysis (MCDA) which combined individual weighting of each criterion with individual scoring for each intervention in each criterion.

**Results:**

By assigning weights, the most important criterion was the quality of evidence (4.01±0.52), and that the comparative cost consequences-non-medical cost was the least important criterion (2.87±1.03). Criteria included disease severity, size of the affected population, comparative effectiveness, type of therapeutic/preventive benefit and cost of intervention, all of which were assigned the same weight of 3.58. After MCDA, the overall value orders for each DPP-4 inhibitor included Sitagliptin (0.45), Linagliptin (0.44), Vildagliptin (0.43), Alogliptin (0.42) and Saxagliptin (0.40).

**Conclusions:**

Based on EVIDEM framework and MCDA, we found that overall value of five DPP-4 inhibitors was similar. It is feasible to use the EVIDEM framework and MCDA in purchasing medicine for Chinese public hospitals.

**Supplementary Information:**

The online version contains supplementary material available at 10.1186/s12913-021-06827-0.

## Background

In Chinese public hospitals, the Pharmacist Management Committee (PMC) makes medicine purchasing decisions based on evidence which made for assessing drug value [1, 2]. With the approval of new drugs and the adjustment of the medical insurance drug catalogue, the PMC of Chinese public Hospital is facing increasing challenges regarding the choice of drug varieties. So far there has not been a canonical method to estimate the value of medicine. The common method to assess the value of drug is to make a systematic review of three dimensions: effectiveness, safety, and economics. However, this method is time consuming and PMC in Chinese public hospitals was asked to make quick decisions. Therefore, evidence sent to PMC is secondary literature or reviews made by rapid assessment which is not systematic or explicit [3]. Meanwhile the assessment mainly focuses on cost-effectiveness analysis and does not systematically consider the multi-dimension value of a drug [3, 4]. The decision-making process is complex, subjective, and requires both systematic and explicit methods to ensure transparency and consistency of the participating factors. There is a need for a systematic and transparent assessment method that meets the requirements for assessment drug in Chinese public hospitals.

Multicriteria decision analysis (MCDA) is a method used to appraise alternatives for the individual that often have conflicting criteria by combining them into a single appraisal [5]. Currently MCDA is applied to health care decision making such as hospital purchasing [6, 7], and helps increase the consistency, transparency and legitimacy of decision making [8]. The Evidence and Value: Impact on Decision Making (EVIDEM) collaboration developed an EVIDEM framework, which aims to estimate medicine systematically, and includes a core model containing 13 criteria that are adaptable to a contextual tool [9] (Tables 1 and 2). EVIDEM framework bridges the MCDA with health technology assessment (HTA) [10]. This study used the 10th edition which is based on 10 years of open source development and its roots are in real-life deliberation and decision.

**Table 1:** EVIDEM Core Model

Domains	Criteria
Need for Intervention	Disease severity
	Size of affected population
	Unmet needs
Comparative Outcomes of Intervention	Comparative effectiveness
	Comparative safety/tolerability
	Comparative patient-perceived health / PRO
Type of Benefit of Intervention	Type of preventive benefit
	Type of therapeutic benefit
Economic Consequences of Intervention	Comparative cost consequences – cost of intervention
	Comparative cost consequences – other medical costs
	Comparative cost consequences – non-medical costs
Knowledge about Intervention	Quality of evidence
	Expert consensus/clinical practice guidelines

**Table 2:** EVIDEM Contextual tool

Domains	Criteria
Normative Contextual Criteria	Mandate and scope of healthcare system
	population priorities and access
	Common goal and specific
	Environmental impact
Feasibility Contextual Criteria	System capacity and appropriate use of intervention
	Political/historical/cultural context
Opportunity Cost	Opportunity costs and affordability

In July 2019, the PMC in a public hospital in Gansu China was asked to make a decision whether to purchase new Dipeptidyl Peptidase-4 (DPP-4) inhibitors to replace the existing ones within a short period of time. The hospital health technology assessment(HTA) group plan to provide evidence by using EVIDEM framework and MCDA to assess the drug. There are currently five DPP-4 inhibitors that are marketed in China: Sitagliptin (approved in 2006), Linagliptin (approved in 2011), Vildagliptin (approved in 2007), Alogliptin (approved in 2013), and Saxagliptin(approved in 2009, [11]). The aim of this study was to appraise DPP-4 inhibitors with the goal of piloting the EVIDEM framework and MCDA to identify the most optimal medication.

## Methods

**Fig. 1:**
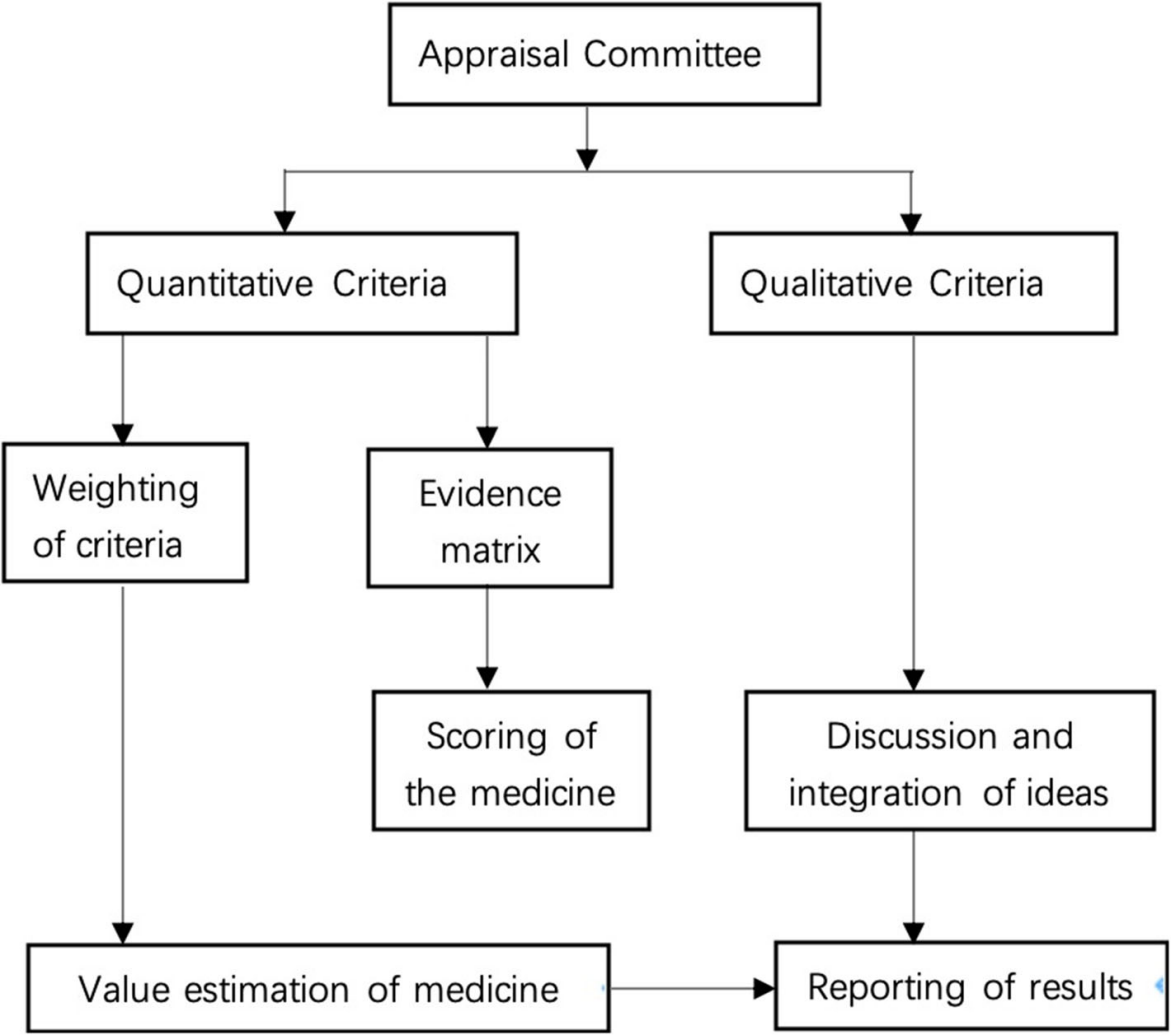
The estimating program

Methodology of the EVIDEM framework was followed for MCDA [8]. MCDA was performed following reports published by International Society for Pharmacoeconomics and Outcomes Research (ISPOR) [12, 13]. The estimating program is shown in Fig. 1.

First, in order to build the EVIDEM evidence matrix of DPP-4 inhibitors, PubMed, Embase, The Cochrane Library and China Biology Medicine disc (CBM) were searched until July 2019. Search terms included the five DPP-4 inhibitors, type 2 diabetes, efficacy, safety, quality of life, epidemiol*/prevalence/incidence, mortality, guidelines, recommendations, clinical practice, patient-reported outcome*/PRO, cost*, econom*, meta-analysis and net meta-analysis (Additional file 2). For the factors that are country-specific (e.g. cost-effectiveness and clinical guidelines), data was only included based on Chinese settings. Placebo was chosen as the comparator. To similarly compare the five DPP-4 inhibitors, meta-analysis comparing the five DPP-4 inhibitors with placebo was first performed. The HTA group analyzed and synthesized the data, meanwhile completed the different criteria of evidence matrix with suitable data.

Second, building an appraisal committee which were comprising two doctors, one nurse, one pharmacist, one health economist and one decision-maker. All six members were already staff at the hospital. Both the doctor and nurse worked in the Endocrinology Institute.

Next, following EVIDEM methodology, the committee assigned weight individually for each criterion in EVIDEM framework according to their importance before it was known which medicines needed to be evaluated. Criterion scored as 5 were considered the most important and those scored as 1 were considered the least important.

Based on the evidence matrix synthesized by the HTA group (Additional file 1), experts assigned a score for each criterion in evidence matrix, individually. The scores for the absolute criteria (those that were not compared to placebo) ranged from 0 to 5, where 0 was the lowest value and 5 was the highest. For relative criteria (those compared to the placebo), the scale ranged from - 5 to 5 to reflect the full range of comparative effects. Meanwhile, criteria in the contextual tool were discussed by committee.

Finally, a value estimate (V) of the intervention was calculated by Excel 2019 based on a linear additive model as a sum of the value contributions (V_*x*_) [or combined normalized weights (W_*x*_) and standardized scores (S_*x*_)] of all (*n*) criteria of the quantitative EVIDEM Core Model. Each estimated value was transformed to a 0-1 scale.

$$ \mathrm{V}=\sum \limits_{x=\mathrm{l}}^{\mathrm{n}}{\mathrm{V}}_x=\sum \limits_{x=\mathrm{l}}^{\mathrm{n}}\left({\mathrm{W}}_x\times {\mathrm{S}}_x\right) $$

## Results

### Criterion weights

Members of the panel committee decided on a mean weight for every criterion as shown in Table 3. Every criterion was of high relevance besides the comparative cost consequences-non-medical cost. The panelists decided the most important criterion to be the quality of evidence. Interestingly, six of the criteria included disease severity, size of the affected population, comparative effectiveness, type of therapeutic/preventive benefit and cost of intervention, which were all assigned the same weight. The type of therapeutic/preventive benefit and non-medical cost showed high variability.

**Table 3:** The mean weight for every criterion

	Mean	SD
Disease severity	3.58	0.75
Size of affected population	3.58	0.75
Unmet needs	3.01	0.55
Comparative effectiveness	3.73	0.82
Comparative safety/tolerability	3.58	0.98
Comparative patient-perceived health / PRO	3.44	0.63
Type of therapeutic benefit	3.58	1.17
Comparative cost consequences – cost of intervention	3.58	0.75
Comparative cost consequences – other medical costs	3.30	0.98
Comparative cost consequences – non-medical costs	2.87	1.03
Quality of evidence	4.01	0.52
Expert consensus/clinical practice guidelines	3.44	0.63

### Scores for the five DPP-4 inhibitors

Mean scores for the five DPP-4 inhibitors are presented in Table 4. Criteria including disease severity, size of the affected population, unmet need, type of therapeutic/preventive benefit, quality of evidence and expert consensus/clinical practice guidelines were evaluated using absolute terms. All of these criteria were given the same scores for all five medicines. The size of affected population showed the highest score and lowest variability in absolute criteria. Disease severity and type of therapeutic benefit followed affected population size. In contrast, the committee did not estimate the type of preventive benefit since there was no data available on this topic. Quality of evidence showed the lowest score based on three meta-analyses evaluated with high bias by the ROBIS tool. Expert consensus/clinical practice guidelines showed the highest variability.

**Table 4:** Mean scores of criterions of five DPP-4 inhibitors

	Saxagliptin		Alogliptin		Sitagliptin		linagliptin		Vildagliptin	
	Mean	SD	Mean	SD	Mean	SD	Mean	SD	Mean	SD
Disease severity	4.00	1.26	4.00	1.26	4.00	1.26	4.00	1.26	4.00	1.26
Size of affected population	4.33	0.52	4.33	0.52	4.33	0.52	4.33	0.52	4.33	0.52
Unmet needs	3.50	0.55	3.50	0.55	3.50	0.55	3.50	0.55	3.50	0.55
Comparative effectiveness	3.50	1.64	4.33	1.03	4.67	0.52	4.33	1.03	3.67	1.51
Comparative safety/tolerability	0.17	0.98	0.33	0.82	0.83	0.75	0.67	1.21	0.67	0.82
Comparative patient-perceived health / PRO	-0.17	0.41	-0.33	0.52	-0.17	0.41	-0.17	0.41	-0.50	0.55
Type of therapeutic benefit	4.00	0.00	4.00	0.00	4.00	0.00	4.00	0.00	4.00	0.00
Type of preventive benefit	/	/	/	/	/	/	/	/	/	/
Comparative cost consequences – cost of intervention	-1.83	1.33	-1.83	1.33	-1.50	0.84	-1.33	0.82	-1.17	0.98
Comparative cost consequences – other medical costs	/	/	/	/	/	/	/	/	/	/
Comparative cost consequences – non-medical costs	-1.83	1.47	-1.67	1.51	-1.33	1.03	-1.50	1.05	-1.33	1.21
Quality of evidence	3.33	0.82	3.33	0.82	3.33	0.82	3.33	0.82	3.33	0.82
Expert consensus/clinical practice guidelines	3.67	1.51	3.67	1.51	3.67	1.51	3.67	1.51	3.67	1.51

Comparing the interventions to placebo, five DPP-4 inhibitors showed greater added value for effectiveness. Sitagliptin showed the highest score for effectiveness. Alogliptin and Linagliptin both had the same scores. Vildagliptin and Saxagliptin both had scores that were less than those of Alogliptin and Linagliptin. In contrast, medicine showed a lower score and higher variability when analyzing comparative safety. Comparative patient-perceived outcomes/PRO received a negative score for all medicines, meaning that the PRO for the medications was worse when compared to the placebo.

In terms of cost, direct costs for Saxagliptin, Alogliptin and Sitagliptin which were purchased by hospital were presented at the hospital price. Linagliptin and Vildagliptin were not located on the hospital purchased list and the mean price used in Chinese nine provinces were calculated. The costs of intervention received a negative score indicating that medicine cost was higher than placebo. There were no data available for other medical costs and thus the committee did not assign scores to these. The committee believed that the medication showed greater non-medical costs with higher variability.

### Estimated value for five DPP-4 inhibitors

**Fig. 2:**
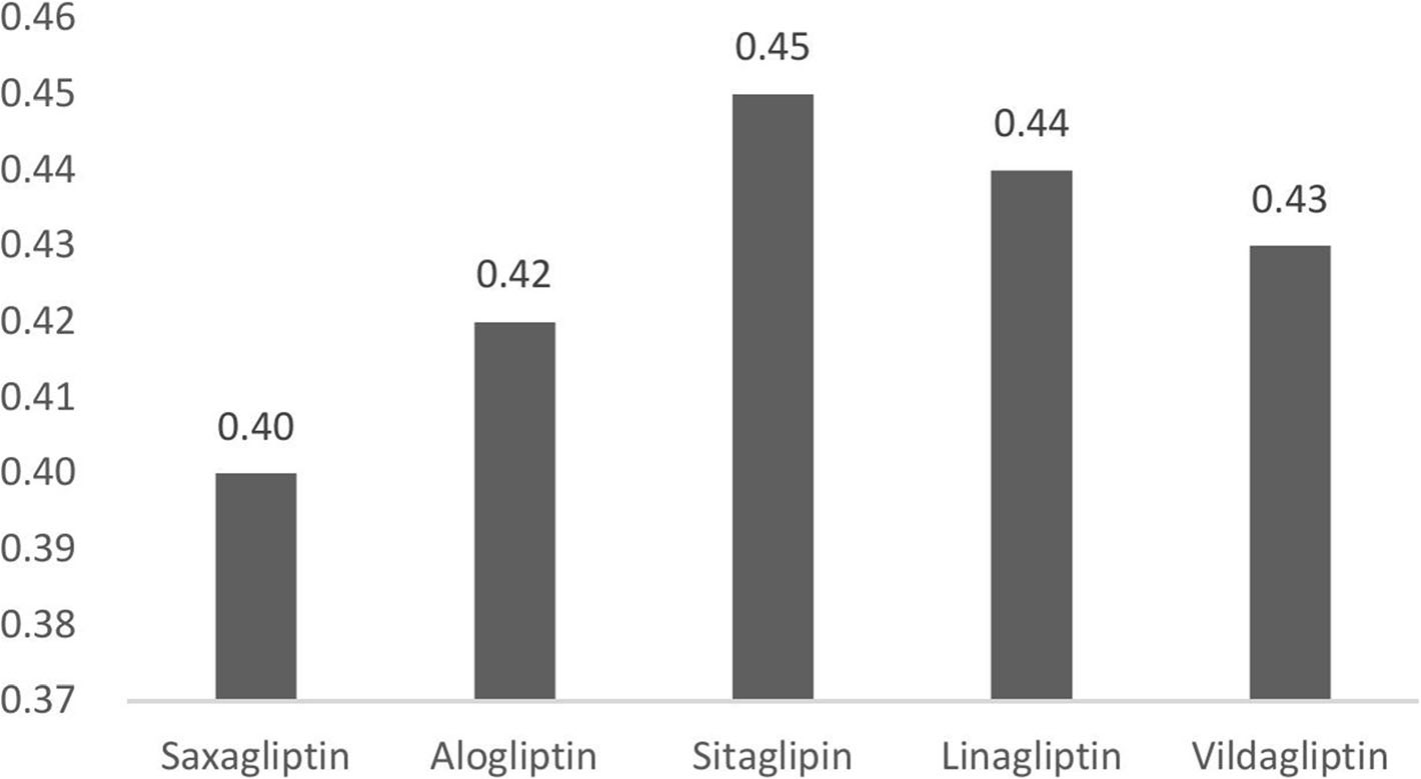
Estimated value for five DPP-4 inhibitors

The overall estimated value that integrated the weights and scores for five DPP-4 inhibitors all showed that the medications had higher added value compared to the placebo (Fig. 2).

## Discussion

In this pilot study Sitagliptin showed the highest value and Saxagliptin received the lowest score. The hospital HTA group gave a value order for decision makers on the hospital PMC, therefore it is feasible to use the EVIDEM framework and MCDA in purchasing medicine for Chinese public hospitals.

To our knowledge, this is the first time that MCDA and EVIDEM frameworks were used in drug purchasing in a Chinese hospital. The MCDA is used in China for the decision-making framework for medical insurance reimbursement and medical device assessment [14, 15]. Our study makes it possible for estimating medicine value with quantitative calculation and includes more comprehensive considerations for the value of medicines by using the EVIDEM framework. Prior to this, the valuation of drug value was qualitative. An HTA group in a hospital in Beijing evaluated DPP-4 inhibitors by using a systematic review that qualitatively described effectiveness, safety and economics [16–18].

The application of EVIDEM and MCDA is still an indirect comparison of drugs. Although there were head-to-head meta-analysis results in terms of safety and effectiveness, no direct comparison data was there for other dimensions of criteria, such as cost. The drug value range was similar with other studies estimating the value by EVIDEM-MCDA. For example, the obinutuzumab for rituximabrefractory indolent non-Hodgkin lymphoma obtained a value of 0.45 in Italy [19]. Interestingly, even though comparative data in the net-meta analysis showed no significant differences between the drugs, value differences still existed [20–24]. Since the weights and scores generated by the committee would inevitably revolve around their own perceptions, experience, training and value judgments, it is critical to have a multi-disciplinary vision.

In applying EVIDEM-MCDA, the participating experts noted that this work was meaningful and inspiring, and the exchange of opinions between them enriched the analysis. However, they also emphasized that the evaluation process requires a good understanding of the amount of evidence provided, and the concepts and terminologies used. In this regard, it is important to emphasize the importance of providing the best understanding. On the other hand, it is worth noting the difference between the specialties of experts that make up the committee. Their weights and scores were inevitably influenced by their own opinions, experience, training and value judgments, thus showing the importance of having such a multidisciplinary vision.

Although this study provides guidance for decision makers, there are still some limitations. Firstly, DPP-4 inhibitors were used a lot in treatment programs of clinical trials such as a DPP-4 inhibitor versus placebo/metformin/other anti-diabetes drugs or a DPP-4 inhibitor/anti-diabetes drugs versus other anti-diabetes drugs [25, 26]. Based on time limitation and feasibility, we chose the placebo as the comparator. There was no consideration from other comparators due to only comparing to the placebo. Secondly, in terms of costs, sufficient data in China was not available since there was little research on Pharmacoeconomics from a Chinese social perspective, especially for the cost of drugs. Due to this, the panelist did not give a score for other-medical costs. Thirdly, although the EVIDEM contained 13 widely identified criteria, other relevant criteria might have been excluded. Fourthly, members of the committee and drug administration in this study limited representation to only one hospital. Thus, the estimated value of the MCDA needs additional interpretation. The MCDA used for complementing pharmacoeconomic analyses lacks the broader perspective, meanwhile it also lacks the discussion basis of ordering health technology [27, 28]. Lastly, we didn’t test the internal consistency of the result, which took the insufficient of the result. This needs to be improved in future research.

## Conclusion

We found that the overall value of five DPP-4 inhibitors was similar. This pilot study assessed the value of the inhibitors and took the value order to help the hospital make decisions regarding drug purchasing using EVIDEM-MCDA.

## Supplementary Information

**Additional file 1.**

**Additional file 2.**

## Data Availability

The datasets used and/or analyzed during the current study are available from the corresponding author on reasonable request.

## Abbreviations

PMC: Pharmacy Management Committee
EVIDEM: Evidence and Value: Impact on Decision Making
MCDA: Multi-Criteria Decision Analysis
HB-HTA: Hospital-Based Health Technology Assessment
HTA: Health Technology Assessment

## References

[1] Zeng Y, Yang M, Luo Y (2004). Standardize the admission procedure of new drugs and optimize the variety of drugs in the hospital. J Pediatr Pharm.

[2] The basic establishment of the hospital drug administration system in China. Med Soc. 2018;31(07):13.

[3] LTH, PM, DZS (2016). Introducing and exploring the method of rapid review on drugs. Clin Med J.

[4] Schlander M (2008). The use of cost-effectiveness by the National Institute for Health and Clinical Excellence (NICE): no(t yet an) exemplar of a deliberative process. J Med Ethics.

[5] Keeney RLRH (1993). Decisions with Multiple Objectives: Preferences and Value Trade-Offs.

[6] Dolan JG (1989). Medical Decision Making Using the Analytic Hierarchy Process:Choice of Initial Antimicrobial Therapy for Acute Pyelonephritis. Med Decis Mak.

[7] van Til JA, Renzenbrink GJ, Dolan JG, Ijzerman MJ (2008). The Use of the Analytic Hierarchy Process to Aid Decision Making in Acquired Equinovarus Deformity. Arch Phys Med Rehabil.

[8] Muhlbacher AC, Kaczynski A (2016). Making Good Decisions in Healthcare with Multi-Criteria Decision Analysis: The Use, Current Research and Future Development of MCDA. Appl Health Econ Health Policy.

[9] Goetghebeur MM, Cellier MS (2018). Can reflective multicriteria be the new paradigm for healthcare decision-making? The EVIDEM journey. Cost Eff Resour Alloc.

[10] Goetghebeur MM, Wagner M, Khoury H, Levitt RJ, Erickson LJ, Rindress D (2008). Evidence and Value: Impact on DEcisionMaking--the EVIDEM framework and potential applications. BMC Health Serv Res.

[11] longzhao C: Inventory of DPP-4 inhibitors listed in China. https://med.sina.com/article_detail_103_2_47380.html 2018.

[12] Thokala P, Devlin N, Marsh K, Baltussen R, Boysen M, Kalo Z, Longrenn T, Mussen F, Peacock S, Watkins J (2016). Multiple Criteria Decision Analysis for Health Care Decision Making--An Introduction: Report 1 of the ISPOR MCDA Emerging Good Practices Task Force. Value Health.

[13] Marsh K, MIJ, Thokala P, Baltussen R, Boysen M, Kalo Z, Lonngren T, Mussen F, Peacock S, Watkins J (2016). Multiple Criteria Decision Analysis for Health Care Decision Making--Emerging Good Practices: Report 2 of the ISPOR MCDA Emerging Good Practices Task Force. Value Health.

[14] Jingsong G, Xiaowei C, Yu X (2018). Study on the evidence-based decision-making framework for reimbursement technologies in view of EVIDEM. Chinese J Health Policy.

[15] Zhilin W (2013). Evaluation Model of Medical Consumables Admission Based on EVIDEM and Its Application. Presented at the 2013 Proceedings of the 14th Academic Annual Meeting of the Chinese Medical Association Medical Engineering Branch Wuhan.

[16] Peng M, Yunchun G, Suodi Z (2017). A rapid assessment of the effectiveness and safety of ritagliptin in the treatment of type 2 diabetes and its economic analysis in China. Chin J Pharmacoepidemiol.

[17] Peng M, Yunchun G, Suodi Z (2016). Vildagliptin in the Treatment of Type 2 Diabetes Mellitus: Health Technology Assessment. Chin J Pharmacoepidemiol.

[18] Peng M, Junwen Z, Hhuilin T (2016). Pharmacoeconomic Systematic Review of Saxagliptin for Type 2 Diabetes. Chinese J Pharm.

[19] Tony M, Wagner M, Khoury H, Rindress D, Papastavros T, Oh P (2011). Bridging health technology assessment (HTA) with multi- criteria decision analyses (MCDA): field testing of the EVIDEM framework for coverage decisions by a public payer in Canada. BMC Health Serv Res.

[20] Cai X, Gao X, Yang W, Chen Y, Zhou L, Zhang S, Han X, Ji L (2016). DPP-4 Inhibitor Treatment in Chinese Type 2 Diabetes Patients: A Meta-Analysis. Diabetes Technol Ther.

[21] Craddy P, Palin HJ, Johnson KI (2014). Comparative effectiveness of dipeptidylpeptidase-4 inhibitors in type 2 diabetes: a systematic review and mixed treatment comparison. Diabetes Ther.

[22] Ling J, Cheng P, Ge L, Zhang DH, Shi AC, Tian JH, Chen YJ, Li XX, Zhang JY, Yang KH (2019). The efficacy and safety of dipeptidyl peptidase-4 inhibitors for type 2 diabetes: a Bayesian network meta-analysis of 58 randomized controlled trials. Acta Diabetol.

[23] Goossen K, Graber S (2012). Longer term safety of dipeptidyl peptidase-4 inhibitors in patients with type 2 diabetes mellitus: systematic review and meta-analysis. Diabetes Obes Metab.

[24] Reaney M, Elash CA, Litcher-Kelly L (2016). Patient Reported Outcomes (PROs) used in recent Phase 3 trials for Type 2 Diabetes: A review of concepts assessed by these PROs and factors to consider when choosing a PRO for future trials. Diabetes Res Clin Pract.

[25] Gao W, Dong J, Liu J, Li Y, Liu F, Yang L, et al. Efficacy and safety of initial combination of DPP-IV inhibitors and metformin versus metformin monotherapy in type 2 diabetes: a systematic review of randomized controlled trials. Diabetes Obes Metab. 16(2):179–85.

[26] Wu S, Chai S, Yang J, Cai T, Zhan S (2017). Gastrointestinal Adverse Events of Dipeptidyl Peptidase 4 Inhibitors in Type 2 Diabetes: A Systematic Review and Network Meta-analysis. Clin Ther.

[27] Tony M, Wagner M, Khoury H, Rindress D, Goetghebeur MM (2011). Bridging health technology assessment (HTA) with multicriteria decision analyses (MCDA): Field testing of the EVIDEM framework for coverage decisions by a public payer in Canada. BMC Health Serv Res.

[28] Baltussen R, Youngkong S, Paolucci F, Niessen L. Multi-criteria decision analysis to prioritize health interventions: Capitalizing on first experiences. Health Policy. 96(3):0–264. 10.1016/j.healthpol.2010.01.009 20206403

