# Supplementary material for: Impact on decision making framework for medicine purchasing in Chinese public hospital decision-making: determining the value of five dipeptidyl peptidase 4 (DPP-4) inhibitors
Source: BMC Health Serv Res. 2021 Aug 12;21:807. doi: 10.1186/s12913-021-06827-0 (PMC8361840; doi:10.1186/s12913-021-06827-0)
Supplement: Supplementary file 1 — Additional file 1. [file 12913_2021_6827_MOESM1_ESM.docx]

| **Comparative Outcomes of Intervention** | | **Saxagliptin** | **Alogliptin** | | **Sitagliptin** | **Linagliptin** | **Vildagliptin** |
| --- | --- | --- | --- | --- | --- | --- | --- |
| **Comparative effectiveness** | - **Cai et al_2016:** Included 30 studies, searched until December 2015 - Inclusion criteria: 1. Clinical trials of DPP-4 inhibitors for monotherapy or combination therapy, 2. Chinese type 2 diabetic population, 3. Study time> 12 weeks - Exclusion criteria: 1. Type 1 diabetes, 2. Study time <12 weeks - Outcomes：HbA1c - Control group: placebo  \|  \| **Saxagliptin** \| **Alogliptin** \| **Sitagliptin** \| **Llinagliptin** \| **Vildagliptin** \| \| --- \| --- \| --- \| --- \| --- \| --- \| \| WMD \| **-0.45*** \| **-0.6*** \| **-0.67*** \| **-0.61*** \| **-0.54*** \| \| 95%CI \| **(-0.54,-0.37)** \| **(-0.69,-0.51)** \| **(-0.93,-0.40)** \| **(-0.75,-0.48)** \| **(-0.78,-0.30)** \|   ***** Significant difference   - **Craddy et al_2014:** Included 82 studies，searched until November 2012, and the study period was 4-104 weeks - Inclusion criteria：1. Clinical trials of DPP-4 inhibitors for monotherapy or combination therapy, 2. Type 2 diabetes with poor glycemic control, 3. Study duration> 12 weeks - Outcomes：HbA1c、Weight - Control group: placebo  \|  \|  \| **Saxagliptin** \| **Alogliptin** \| **Sitagliptin** \| **Llinagliptin** \| **Vildagliptin** \| \| --- \| --- \| --- \| --- \| --- \| --- \| --- \| \| HbA1c \| WMD \| **-0.593*** \| **-0.797*** \| **-0.788*** \| **-0.734*** \| **-0.60*** \| \|  \| 95%CI \| **(-0.811,-0.375)** \| **(-0.943,-0.651)** \| **(-0.954,-0.622)** \| **(-0.880,-0.588)** \| **(-0.80,-0.40)** \| \| HbA1c \| OR \| **1..909*** \| **3.157*** \| **3.934*** \| **2.772*** \| **4.105*** \| \| ＜7% \| 95%CI \| **(1.03,3.56)** \| **(1.87,5.49)** \| **(2.20,7.05)** \| **(1.74,4.41)** \| **(1.95,8.63)** \| \| Weight \| WMD \| **NA** \| **0.049** \| **0.717*** \| **0.431*** \| **1.25*** \| \| 95%CI \| **（-0.53,0.62）** \| **（0.37,1.06）** \| **(0.004,0.86)** \| **(0.47,2.03)** \|   ***** Significant difference   - Bessie's meta-analysis revealed no difference between the five DPP-4 inhibitors in improving baseline HbA1c and weight, and in the proportion of patients who achieved HbA1c <7%. | ☐ 5 Much better than comparator | ☐ 5 Much better than comparator | | ☐ 5 Much better than comparator | ☐ 5 Much better than comparator | ☐ 5 Much better than comparator |
|  |  | ☐ 4 | ☐ 4 | | ☐ 4 | ☐ 4 | ☐ 4 |
|  |  | ☐ 3 | ☐ 3 | | ☐ 3 | ☐ 3 | ☐ 3 |
|  |  | ☐ 2 | ☐ 2 | | ☐ 2 | ☐ 2 | ☐ 2 |
|  |  | ☐ 1 | ☐ 1 | | ☐ 1 | ☐ 1 | ☐ 1 |
|  |  | ☐ 0 No difference | ☐ 0 No difference | | ☐ 0 No difference | ☐ 0 No difference | ☐ 0 No difference |
|  |  | ☐ -1 | ☐ -1 | | ☐ -1 | ☐ -1 | ☐ -1 |
|  |  | ☐ -2 | ☐ -2 | | ☐ -2 | ☐ -2 | ☐ -2 |
|  |  | ☐ -3 | ☐ -3 | | ☐ -3 | ☐ -3 | ☐ -3 |
|  |  | ☐ -4 | ☐ -4 | | ☐ -4 | ☐ -4 | ☐ -4 |
|  |  | ☐ -5 Much worse than comparator | ☐ -5 Much worse than comparator | | ☐ -5 Much worse than comparator | ☐ -5 Much worse than comparator | ☐ -5 Much worse than comparator |
| **Comparative Outcomes of Intervention** | | **Saxagliptin** | **Alogliptin** | | **Sitagliptin** | **Linagliptin** | **Vildagliptin** |
| **Comparative safety/tolerability** | - **Ling et al_2019：**Included 58 studies，searched until April 2018，the study period was 4-54 weeks - Included studies：1. RCTs, 2. Type 2 diabetes patients, - Outcomes：Hypoglycemia, Diarrhea, Hypersensitivity, Upper respiratory infection (URTI), Liver and Kidney toxicity - Control group: placebo  \|  \|  \| **Saxagliptin** \| **Alogliptin** \| **Sitagliptin** \| **Llinagliptin** \| **Vildagliptin** \| \| --- \| --- \| --- \| --- \| --- \| --- \| --- \| \| Hypoglycemia \| HR \| 2.12 \| 2.74 \| 1.19 \| 0.90 \| 1.05 \| \| 95%CI \| (0.86,5.51) \| (0.59,14.49) \| (0.66,2.11) \| (0.54，1.38) \| (0.47,2.15) \| \| Diarrhea \| HR \| 1.54 \| NA \| 1.37 \| NA \| 1.09 \| \| 95%CI \| (0.42,6.22) \| (0.46,3.89) \| (0.27,3.93) \| \| URTI \| HR \| NA \| NA \| **0.72*** \| 1.19 \| 1.04 \| \| 95%CI \| (2.93,1.15) \| (0.41,3.00) \| (0.22,4.56) \| \| Liver and Kidney toxicity \| HR \| NA \| NA \| 1.06 \| 0.74 \| 1.70 \| \| 95%CI \| (0.05,28.36) \| (0.05,10.27) \| (0.03,15.10) \| \| Hypersensitivity \| HR \| NA \| NA \| NA \| 2.41 \| 4.94 \| \| 95%CI \| (0.53,92.28) \| (0.03,12.58)) \|   ***** Significant difference   - SUCRA tips: Sitagliptin 50mg QD was the least likely to have URTI; Liglitintin 5mg QD was the least likely to have an adverse reaction - **Gooben et al_2012：**Included 67 studies，searched until October 2011, and the study time was> 18 weeks - Included studies:1. RCTs，2. type 2 diabetes, - Outcomes：Any adverse event (AE), serious adverse event, adverse event that interrupted treatment - Control group: placebo  \|  \|  \| **Saxagliptin** \| **Alogliptin** \| **Sitagliptin** \| **Llinagliptin** \| **Vildagliptin** \| \| --- \| --- \| --- \| --- \| --- \| --- \| --- \| \| AE \| RR \| 1.04 \| 1.01 \| 1.03 \| 1.01 \| 1.00 \| \| 95%CI \| (0.99,1.10) \| (0.93,1.09) \| (0.98,1.07) \| (0.95,1.08) \| (0.96.1.04) \| \| serious AE \| RR \| 1.04 \| 1.3 \| 1.02 \| 0.71 \| 0.89 \| \| 95%CI \| (0.79,1.39) \| (0.76,2.22) \| (0.81,1.28) \| (0.47,1.06) \| (0.71,1.11) \| \| AE that interrupted treatment \| RR \| 1.35 \| 1.15 \| 1.00 \| 0.85 \| 1.01 \| \| 95%CI \| (0.90,2.03) \| (0.57,2.32) \| (0.75,1.33) \| (0.55,1.30) \| (0.77,1.33) \|  - For most system organs, adverse events with 5 DPP-4 inhibitors were no different from placebo. - Fewer than 1% of patients died during treatment and only about 40% of studies reported deaths. - No increase or decrease in cardiovascular risk. - Warning: The FDA warns that saxagliptin, lidagliptin, alogliptin, and sitagliptin can cause joint pain. | ☐ 5 Much better than comparator | ☐ 5 Much better than comparator | | ☐ 5 Much better than comparator | ☐ 5 Much better than comparator | ☐ 5 Much better than comparator |
|  |  | ☐ 4 | ☐ 4 | | ☐ 4 | ☐ 4 | ☐ 4 |
|  |  | ☐ 3 | ☐ 3 | | ☐ 3 | ☐ 3 | ☐ 3 |
|  |  | ☐ 2 | ☐ 2 | | ☐ 2 | ☐ 2 | ☐ 2 |
|  |  | ☐ 1 | ☐ 1 | | ☐ 1 | ☐ 1 | ☐ 1 |
|  |  | ☐ 0 No difference | ☐ 0 No difference | | ☐ 0 No difference | ☐ 0 No difference | ☐ 0 No difference |
|  |  | ☐ -1 | ☐ -1 | | ☐ -1 | ☐ -1 | ☐ -1 |
|  |  | ☐ -2 | ☐ -2 | | ☐ -2 | ☐ -2 | ☐ -2 |
|  |  | ☐ -3 | ☐ -3 | | ☐ -3 | ☐ -3 | ☐ -3 |
|  |  | ☐ -4 | ☐ -4 | | ☐ -4 | ☐ -4 | ☐ -4 |
|  |  | ☐ -5 Much worse than comparator | ☐ -5 Much worse than comparator | | ☐ -5 Much worse than comparator | ☐ -5 Much worse than comparator | ☐ -5 Much worse than comparator |
| **Comparative Outcomes of Intervention** | | **Saxagliptin** | **Alogliptin** | | **Sitagliptin** | **Linagliptin** | **Vildagliptin** |
| **Comparative patient-perceived health / PRO** | - There were currently no studies on PRO for DPP-4 inhibitors. - Gooben et al_2012：   Compared with the placebo, patients with vitagliptin experienced fatigue RR 1.57(95%CI 1.09-2.77), with a significant difference   - Craddy et al_2014: Hypoglycemic  \|  \| **Saxagliptin** \| **Alogliptin** \| **Sitagliptin** \| **Llinagliptin** \| **Vildagliptin** \| \| --- \| --- \| --- \| --- \| --- \| --- \| \| RR \| 0.257 \| 0.949 \| 0.924 \| 0.311 \| NA \| \| 95%CI \| (0.49,13.13) \| (0.06,15.45) \| (0.23,3.77) \| (0.04,2.55) \|   ***** Significant difference   - Bayesian meta-analysis suggested that there was no difference in the risk of hypoglycemic events among the five DPP-4 inhibitors | ☐ 5 Much better than comparator | ☐ 5 Much better than comparator | ☐ 5 Much better than comparator | | ☐ 5 Much better than comparator | ☐ 5 Much better than comparator |
|  |  | ☐ 4 | ☐ 4 | ☐ 4 | | ☐ 4 | ☐ 4 |
|  |  | ☐ 3 | ☐ 3 | ☐ 3 | | ☐ 3 | ☐ 3 |
|  |  | ☐ 2 | ☐ 2 | ☐ 2 | | ☐ 2 | ☐ 2 |
|  |  | ☐ 1 | ☐ 1 | ☐ 1 | | ☐ 1 | ☐ 1 |
|  |  | ☐ 0 No difference | ☐ 0 No difference | ☐ 0 No difference | | ☐ 0 No difference | ☐ 0 No difference |
|  |  | ☐ -1 | ☐ -1 | ☐ -1 | | ☐ -1 | ☐ -1 |
|  |  | ☐ -2 | ☐ -2 | ☐ -2 | | ☐ -2 | ☐ -2 |
|  |  | ☐ -3 | ☐ -3 | ☐ -3 | | ☐ -3 | ☐ -3 |
|  |  | ☐ -4 | ☐ -4 | ☐ -4 | | ☐ -4 | ☐ -4 |
|  |  | ☐ -5 Much worse than comparator | ☐ -5 Much worse than comparator | ☐ -5 Much worse than comparator | | ☐ -5 Much worse than comparator | ☐ -5 Much worse than comparator |
| **Type of Benefit of Intervention** | |  | | | | | |
| Type of therapeutic benefit | - At the patient level: the purpose of treatment is to control the patient's blood sugar | ☐ 5 Cure  ☐ 4  ☐ 3  ☐ 2  ☐ 1  ☐ 0 No therapeutic benefit | | | | | |

| **Economic Consequences of Intervention** | | **Saxagliptin** | **Alogliptin** | **Sitagliptin** | **Linagliptin** | **Vildagliptin** | |
| --- | --- | --- | --- | --- | --- | --- | --- |
| cost of intervention | \|  \| Saxagliptin* \| Alogliptin* \| Sitagliptin* \| Llinagliptin# \| Vildagliptin# \| \| --- \| --- \| --- \| --- \| --- \| --- \| \| Dose \| 5mg \| 25mg \| 100mg \| 5mg \| 50mg \| \| Frequency \| QD \| QD \| QD \| QD \| BID \| \| Average price per piece（yuan） \| 7.95 \| 8.07 \| 7.52 \| 8.44 \| 4.26 \| \| Average daily drug cost per patient（yuan） \| 7.95 \| 8.07 \| 7.52 \| 8.44 \| 8.52 \|   *Hospital purchasing price，#Average price of nine provinces | ☐ 5 Substantial savings | ☐ 5 Substantial savings | ☐ 5 Substantial savings | ☐ 5 Substantial savings | ☐ 5 Substantial savings | |
|  |  | ☐ 4 | ☐ 4 | ☐ 4 | ☐ 4 | ☐ 4 | |
|  |  | ☐ 3 | ☐ 3 | ☐ 3 | ☐ 3 | ☐ 3 | |
|  |  | ☐ 2 | ☐ 2 | ☐ 2 | ☐ 2 | ☐ 2 | |
|  |  | ☐ 1 | ☐ 1 | ☐ 1 | ☐ 1 | ☐ 1 | |
|  |  | ☐ 0 No change in spending | ☐ 0 No change in spending | ☐ 0 No change in spending | ☐ 0 No change in spending | ☐ 0 No change in spending | |
|  |  | ☐ -1 | ☐ -1 | ☐ -1 | ☐ -1 | ☐ -1 | |
|  |  | ☐ -2 | ☐ -2 | ☐ -2 | ☐ -2 | ☐ -2 | |
|  |  | ☐ -3 | ☐ -3 | ☐ -3 | ☐ -3 | ☐ -3 | |
|  |  | ☐ -4 | ☐ -4 | ☐ -4 | ☐ -4 | ☐ -4 | |
|  |  | ☐ -5 Substantial additional expenditures | ☐ -5 Substantial additional expenditures | ☐ -5 Substantial additional expenditures | ☐ -5 Substantial additional expenditures | ☐ -5 Substantial additional expenditures | |
| **Economic Consequences of Intervention** | | **Saxagliptin** | **Alogliptin** | **Sitagliptin** | **Linagliptin** | **Vildagliptin** | |
| non-medical costs | - There was no economic studies of DPP-4 inhibitors versus placebos in Chinese society or hospital perspective - Gao_2016：The society perspective - Initiation status: uncomplicated type 2 diabetes in 50-60 years old - Intervention group vs control group: DPP-4 inhibitor combined with metformin vs metformin - Model type: Markov model - Cycle time: 1.5 years, half-cycle correction - Discount: 3% - Threshold: 2014 GDP <threshold <3 times GDP per capita - Sensitivity analysis: one-dimensional sensitivity analysis  \|  \| Saxagliptin+ metformin \| Alogliptin+ metformin \| Sitagliptin+ metformin \| Llinagliptin+ metformin \| Vildagliptin+ metformin \| \| --- \| --- \| --- \| --- \| --- \| --- \| \| Cost \| 163404.43 \| 158987.6 \| **157808.48** \| 163634.32 \| 162048.91 \| | ☐ 5 Substantial savings | ☐ 5 Substantial savings | ☐ 5 Substantial savings | ☐ 5 Substantial savings | ☐ 5 Substantial savings | |
|  |  | ☐ 4 | ☐ 4 | ☐ 4 | ☐ 4 | ☐ 4 | |
|  |  | ☐ 3 | ☐ 3 | ☐ 3 | ☐ 3 | ☐ 3 | |
|  |  | ☐ 2 | ☐ 2 | ☐ 2 | ☐ 2 | ☐ 2 | |
|  |  | ☐ 1 | ☐ 1 | ☐ 1 | ☐ 1 | ☐ 1 | |
|  |  | ☐ 0 No change in spending | ☐ 0 No change in spending | ☐ 0 No change in spending | ☐ 0 No change in spending | ☐ 0 No change in spending | |
|  |  | ☐ -1 | ☐ -1 | ☐ -1 | ☐ -1 | ☐ -1 | |
|  |  | ☐ -2 | ☐ -2 | ☐ -2 | ☐ -2 | ☐ -2 | |
|  |  | ☐ -3 | ☐ -3 | ☐ -3 | ☐ -3 | ☐ -3 | |
|  |  | ☐ -4 | ☐ -4 | ☐ -4 | ☐ -4 | ☐ -4 | |
|  |  | ☐ -5 Substantial additional expenditures | ☐ -5 Substantial additional expenditures | ☐ -5 Substantial additional expenditures | ☐ -5 Substantial additional expenditures | ☐ -5 Substantial additional expenditures | |
| **Knowledge about Intervention** | |  | | | | |  |
| Knowledge about Intervention | - Meta-analysis：The ROBIS tool was used to evaluate the methodological quality of the literature. One of the four literatures was low bias, one was judged to be high bias because the bias of the original study was not reported, and two were judged to be high bias because they did not describe whether the literature was collected through other channels. - Economic literature: Using the SIGN checklist to evaluate the quality of the literature methodology for high-quality research. | ☐ 5 Highly relevant and valid | | | | |  |
|  |  | ☐ 4 | | | | |  |
|  |  | ☐ 3 | | | | |  |
|  |  | ☐ 2 | | | | |  |
|  |  | ☐ 1 | | | | |  |
|  |  | ☐ 0 Not relevant and/or invalid | | | | |  |
| clinical practice guidelines | - *China Type 2 Diabetes Prevention Guidelines* recommended by the Diabetes Branch of the Chinese Medical Association in 2017. There was no recommendation level. | ☐ 5 Strong recommendation for intervention above all other alternatives | | | | |  |
|  |  | ☐ 4 | | | | |  |
|  |  | ☐ 3 | | | | |  |
|  |  | ☐ 2 | | | | |  |
|  |  | ☐ 1  ☐ 0 Not recommended | | | | |  |
|  |  |  | | | | |  |

| **Need for Intervention** | |  |
| --- | --- | --- |
| Disease severity | - Type 2 diabetes is caused by pancreatic beta-cell dysfunction or insulin resistance in the target organ. The pancreas, liver, skeletal muscle, kidney, brain, small intestine, and adipose tissue are involved in the development of type 2 diabetes. In the hyperglycemia environment, large blood vessels, micro vessels, nerves, etc. will all be affected, which will harm the heart, kidneys, eyes and other organs. Type 2 diabetes increases the risk of death from coronary heart disease, ischemic stroke, and other vascular diseases. Cardiovascular disease is the biggest cause of type 2 diabetes morbidity and mortality. Compared with people without diabetes, patients with type 2 diabetes have a 15% increase in all-cause mortality. | ☐ 5 Very severe |
|  |  | ☐ 4 |
|  |  | ☐ 3 |
|  |  | ☐ 2 |
|  |  | ☐ 1 |
|  |  | ☐ 0 Not severe |
|  |  |  |
| Size of affected population | - According to data released by the International Diabetes Federation, the number of diabetic patients in China reached 114 million in 2017, more than 90% of whom had type 2 diabetes, and it is expected to increase to 642 million by 2040. Data from Gansu Province show that the prevalence of type 2 diabetes in Gansu Province in 2014 was 9.7%. | ☐ 5 Common disease |
|  |  | ☐ 4 |
|  |  | ☐ 3 |
|  |  | ☐ 2 |
|  |  | ☐ 1 |
|  |  | ☐ 0 Very rare disease |
|  |  |  |
| Unmet needs | - **Metformin** is associated with a decrease in vitamin B12 and is banned in patients with moderate to severe chronic kidney disease. **Sulfonylureas** increase weight and the risk of hypoglycemia, and are associated with adverse consequences of cardiovascular disease. **Thiazolidinediones** has been controversial, troglitazone is withdrawn due to liver toxicity, rosiglitazone is rarely used due to adverse cardiovascular effects, pioglitazone treatment is associated with fractures; patients with a history of chronic pancreatitis or pancreatic cancer have disabled GLP-1 receptor agonism Agent. The main side effect of **SGLT-2 inhibitor** is urinary tract or reproductive tract infection, which is less effective in patients with moderate to severe renal insufficiency. It should not be used when eGFR is less than 30mL / min / 1 · 73m^2^. SGLT-2 inhibitor Associated with ketoacidosis at normal blood sugar, discontinued during acute illness and hospitalization. **Insulin** is associated with an increased risk of hypoglycemia, especially in the elderly. - The main difficulties with current medications for hypoglycemic treatment are clinical inertia and treatment non-compliance. | ☐ 5 Many unmet needs |
|  |  | ☐ 4 |
|  |  | ☐ 3 |
|  |  | ☐ 2 |
|  |  | ☐ 1 |
|  |  | ☐ 0 No unmet needs |
|  |  |  |
